# A single-arm phase II trial of combined chemotherapy with S-1, oral leucovorin, and bevacizumab in heavily pre-treated patients with metastatic colorectal cancer

**DOI:** 10.1186/s12885-015-1606-1

**Published:** 2015-08-27

**Authors:** Kazuhisa Yamaguchi, Hiroya Taniguchi, Azusa Komori, Yukiya Narita, Sohei Nitta, Motoo Nomura, Shigenori Kadowaki, Daisuke Takahari, Takashi Ura, Masashi Andoh, Kei Muro, Keita Mori, Yoshinori Igarashi

**Affiliations:** 1 Department of Clinical Oncology, Aichi Cancer Center Hospital, 1-1 Kanokoden, Chikusa-ku, Nagoya, 464-8681 Aichi Japan; 2 Division of Gastroenterology and Hepatology, Department of Internal Medicine, Toho University Omori Medical Center, 6-11-1 Omorinishi, Ota-ku, 143-8541 Tokyo Japan; 3 Division of Clinical Research Promotion Office, Depertment of Clinical, Research Support Center, Shizuoka Cancer Center, 1007 Shimonagakubo, Nagaizumi–cho, Sunto-gun, 411-8777 Shizuoka Japan

**Keywords:** Metastatic colorectal cancer, S-1, Bevacizumab, Leucovorin, KRAS, BRAF, Chemotherapy

## Abstract

**Background:**

The mean 5–6-month survival after failed standard chemotherapy for metastatic colorectal cancer (mCRC) necessitates more effective treatments for refractory mCRC. For untreated mCRC, S-1 + oral leucovorin (SL) therapy offers promising results without severe toxicity. The ML18147 trial demonstrated that bevacizumab (Bev) prolongs overall survival after mCRC progression. We conducted a single-centre phase-II trial to evaluate the safety and efficacy of SL/Bev combination chemotherapy as mCRC salvage therapy.

**Methods:**

Major eligibility criteria were confirmed adenocarcinoma diagnosis; age >20 years; Eastern Cooperative Oncology Group performance status, 0–2; and progression after administration/intolerance of/to approved drugs for mCRC. (5-FU, oxaliplatin, irinotecan, Bev, and anti-EGFR antibody, if KRAS wild-type). S-1 (80–120 mg/body) and leucovorin (25 mg) were orally administered in a 1-week-on/1-week-off schedule. Bev (5 mg/kg) was administered on day 1 of every 2-week cycle. The primary endpoint was disease control rate (DCR).

**Results:**

A total of 31 patients were enrolled. DCR was 65 % [95 % confidence interval (CI), 48–100 %] and the response rate was 7 % (95 % CI, 0.7–22 %). One patient showing partial response to SL/Bev had a *BRAF*-mutant tumor. Median progression-free survival and overall survivals were 5.3 [95 % CI, 2.1–9.3] and 9.9 [95 % CI, 7.4–NA] months, respectively. The most-frequent grade-3/4 adverse events were mucositis (26 %) and diarrhea (11 %), which were manageable by dose reduction/interruption.

**Conclusions:**

SL/Bev showed impressive activity in refractory mCRC and was tolerable, suggesting its potential as an alternative chemotherapy for refractory mCRC.

**Trial registration:**

This study has been registered in University Hospital Medical Information Network (UMIN) Clinical Trials Registry (IDUMIN000009083) on 11 October 2012.

**Electronic supplementary material:**

The online version of this article (doi:10.1186/s12885-015-1606-1) contains supplementary material, which is available to authorized users.

## Background

Systemic chemotherapy for metastatic colorectal cancer (mCRC) has improved remarkably in recent years, currently allowing most mCRC patients to survive for >2 years. Standard treatments for patients with mCRC include chemotherapy regimens based on cytotoxic agents, such as fluoropyrimidine, irinotecan, oxaliplatin and the vascular endothelial growth factor (VEGF) antibody bevacizumab (Bev), with the addition of epithelial growth factor receptor (EGFR) antibodies cetuximab and panitumumab for RAS wild-type patients [1, 2]. An additional file shows standard therapies for metastatic colorectal cancer in the various setting [see Additional file 1]. However, after failure of standard chemotherapy, the average survival rate is only 5–6 months. Therefore, there is a need for more effective treatment for patients with refractory mCRC who maintain a relatively good performance status (PS) and are willing to receive further treatment.

S-1 (TS-1; Taiho Pharmaceutical Co. Ltd., Tokyo, Japan) is an oral fluoropyrimidine anti-cancer agent that combines tegafur as the effector drug with the 2 modulators gimeracil and oteracil. Tegafur is a pro-drug of 5-fluorouracil (5-FU), gimeracil is an inhibitor of dihydropyrimidine dehydrogenase (DPD) which maintains the serum 5-FU level. Oteracil inhibits the gastrointestinal toxicity of 5-FU. Several phase-III trials on mCRC have demonstrated S-1 can be a substitute for infusional 5-FU [3, 4]. Moreover, a phase-II study demonstrated promising S-1 activity in heavily pre-treated mCRC patients, including those treated with 5-FU [5].

Leucovorin (LV) is a well-known enhancer of 5-FU activity by inhibiting thymidylate synthase (TS) [6]. Concomitant 5-FU + LV therapy is used worldwide to treat patients with mCRC as either first-line or adjuvant therapy. In addition, oral tegafur/uracil (UFT) plus LV combination therapy has demonstrated similar clinical efficacy to that of intravenous 5-FU and LV, and it is associated with improved convenience of care because an infusion pump is not required [7]. On the other hand, little data is available on S-1 plus oral LV (SL) combination therapy. Subsequently, Koizumi et al. conducted a phase-II study of SL treatment in patients with previously untreated mCRC, the results of which were promising [8]. On the basis of these results, SL therapy is expected to yield high anti-tumour activity compared with S1 monotherapy in cases of refractory mCRC.

Bev is a humanized monoclonal antibody that inhibits VEGF and has demonstrated activity both as first-line and second-line therapy for mCRC in combination with fluoropyrimidine with or without irinotecan or oxaliplatin [9–11]. However, the role of Bev in third-line or later-line therapy of mCRC remains unclear. The continuation of Bev beyond disease progression after first-line therapy has been demonstrated to improve progression-free survival (PFS) and overall survival (OS) in large phase-III trials [12, 13]. These results suggest that Bev therapy after disease progression may have a clinical benefit even in refractory patients treated with Bev.

To test these hypotheses, we conducted a single-centre phase-II trial to assess the efficacy and safety of SL + Bev (SL/Bev) as a salvage therapy in patients with mCRC in whom prior chemotherapy with 5-FU, oxaliplatin, irinotecan, Bev and anti-EGFR antibodies has failed.

## Methods

### Patient eligibility

Major eligibility criteria were mCRC progression after administration/intolerance of/to 5-fluouracil, oxaliplatin, irinotecan and Bev, as well as anti-EGFR antibodies for patient with *KRAS* wild-type tumours (*KRAS* mutational status was detected in codons 12 and 13 using Cycleave polymerase chain reaction technique). Other eligibility criteria were as follows; age ≥20 years; histologically confirmed adenocarcinoma of the colon/rectum; at least 1 measurable lesion according to Response Evaluation Criteria in Solid Tumours (RECIST, version 1.1); Eastern Cooperative Oncology Group (ECOG) Performance Status (PS) 0–2 [14]; ability to take drugs orally; no prior S-1 therapy; adequate bone-marrow function (a neutrophil count of ≥1500/mm^3^, a haemoglobin level of ≥8 g/dl, a platelet count of ≥75,000/mm^3^), adequate liver function (a serum total bilirubin level of ≤1.5 mg/dl, serum aspartate aminotransferase and alanine aminotransferase levels of ≤200 IU/l), and adequate renal function (a serum creatinine level of ≤1.2 mg/dl and creatinine clearance ≥50 ml/min). Major exclusion criteria included prior surgery, chemotherapy/radiotherapy within 2 weeks of entering the trial, uncontrolled comorbidities, active infection, symptomatic brain metastases and severe ascites/pleural effusion. The study protocol was approved by the ethics review committee of Aichi Cancer Center Hospital and informed consent was obtained before enrolment from all patients. The study protocol was registered at the University Hospital Medical Information Network (UMIN) Clinical Trials Registry (protocol ID UMIN000009083).

### Study treatment

S-1 [80 mg/day for body surface area (BSA) <1.25 m^2^; 100 mg/day for 1.25 ≤ BSA < 1.50 m^2^; and 120 mg/day for BSA ≥1.50 m^2^] and LV (50 mg/day, fixed dose) were orally administered twice daily for 1 week followed by a 1-week rest. Bev (5 mg/kg) was administered as an intravenous infusion over 30 min on day 1 of every 2-week cycle. If patients had no infusion reaction, the infusion time was shortened to 15 min. In the event of grade-4 neutropenia or thrombocytopenia, grade-3 diarrhea or stomatitis, and febrile neutropenia as well as depending on the degree of toxicity in each patient, S-1 dose was decreased by 1 level in the subsequent cycle. LV and Bev doses were not decreased. Treatments were continued until disease progression, unacceptable toxicity, or withdrawal of consent. The treatment was discontinued if treatment cycle was delayed for >28 days or dose reduction was required after a second step of reduction. Post-study anti-cancer treatment was allowed on this protocol.

### Assessments of efficacy and toxicity

Treatment response was evaluated in accordance with the Response Evaluation Criteria in Solid Tumours (RECIST version 1.1). The evaluation was performed at baseline and every 8 weeks by computed tomography (CT). A baseline CT scan was done within 4 weeks of starting treatment. Best overall response was assessed by a blinded review by an independent committee, which included two radiologists. Response was not confirmed with repeat scans in the study. The incidence and severity of adverse events were graded using the National Cancer Institute Common Toxicity Criteria version 4.0. Quality of life (QOL) was assessed using EQ5D scores from a patient report at baseline and every 2 weeks thereafter. EQ5D is a standardised measure of the course of health processes. It consists of 5 descriptive questions regarding dimensions of morbidity, self-care, usual activities, pain/discomfort and anxiety/depression. Each dimension has 3 levels of response indicating the severity of a patient’s problems.

### Study endpoints

The primary endpoint of this study was the disease control rate [DCR: complete remission (CR) + partial remission (PR) + stable disease (SD)], as assessed by the independent review committee. The secondary endpoints were PFS, OS, safety and QOL. The study was conducted according to the intention-to-treatment principle (ITT). PFS and OS were calculated as the time between the first day of treatment and the day of proven disease progression or death from any cause. Other causes of events without disease progression were defined as censored. Survival curves were estimated using the Kaplan-Meier method. Statistical analyses were performed using R software version 2.13.2 (R Project for Statistical Computing, Vienna, Austria).

### Statistical analyses

A one sample binomial design was used to determine the sample size. The estimates were based on DCR of previous two trials comparing new drugs and best supportive care (BSC). DCR in the trial of regorafenib and TAS102 were 41 and 43.8 %, while the DCR of BSC group in these trials were 15 and 10.5 %, respectively [15, 16]. Therefore, we hypothesised it would be beneficial if DCR was at least 44 % with this therapy, while under 22 % would be the lower limit of interest. On the basis of this assumption, the required sample size was calculated to be 28 patients according to a null hypothesis of a DCR of ≤22 % versus the alternative hypothesis of a DCR of >44 %, with 80 % power and 0.05 alpha value (one-sided). Considering that some patients may become ineligible after enrolment, the target sample size was determined to be at least 30 patients.

## Results

### Patient characteristics

Between October 2012 and November 2013, a total of 31 patients with mCRC were enrolled in this study at the Aichi Cancer Center Hospital in Japan. Baseline characteristics of the patients are summarized in Table 1. The median age was 69 years (range 37–86 years, interquartile range 61–73 years). Twenty-one patients were male and 10 were female. Most patients had ECOG PS of 0 or 1 (29 patients; 94 %). The primary site was the colon in 25 patients (81 %) and rectum in 6 (19 %). *KRAS* and *BRAF* mutations were present in 13 (37 %) and two patients (7 %), respectively. Twenty-six patients (84 %) had undergone primary tumour resections and 7 (22 %) had been treated with >4 lines of chemotherapy prior to inclusion in the study.

**Table 1:** Baseline patient characteristics (*n* = 31)

Characteristics	*N* (%)
Age (years)	
Median	69
Range, interquartile range	(37–86, 61–73)
Gender	
Male	21 (68)
Female	10 (32)
ECOG performance status	
0	13 (42)
1	16 (52)
2	2 (6)
Primary tumor location	
Colon	25 (81)
Rectum	6 (19)
Primary resected	
Yes	26 (84)
No	5 (16)
No of metastatic site,	
1	10 (32)
2	9 (29)
≥ 3	12 (39)
Metastatic sites	
Liver	22 (71)
Lung	17 (55)
Lymph nodes	13 (42)
Peritoneum	9 (29)
Other	4 (13)
*KRAS* mutation	
Yes	13 (37)
No	18 (63)
*BRAF* mutation	
Yes	2 (6)
No	26 (84)
Unknown	3 (10)
Number of lines prior therapy	
2	8 (19)
3	16 (59)
≥ 4	7 (22)
Median CEA level ng/ml (range, interquartile range)	116 (0–42,230, 31–350)
Median CA19-9 level ng/ml (range, interquartile range)	222 (5–9820, 38–965)

*ECOG* Eastern Cooperative Oncology Group, *CEA* serum carcinoembryonic antigen, *CA19-9* carbohydrate antigen 19–9

### Treatment results and efficacy

**Fig. 1:**
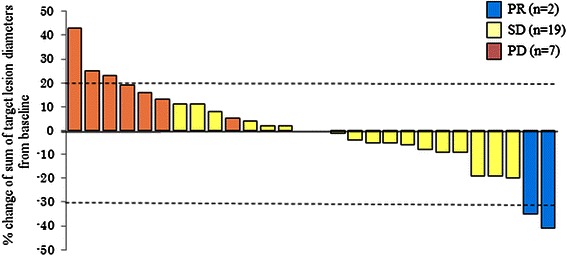
Waterfall plots of best overall percent change from baseline in sum of target lesion diameters. Evaluation was assessed by the central review committee. (evaluable patients only, *n* = 28). Abbreviations; PR, partial response; SD, stable disease; PD, progressive disease

**Fig. 2:**
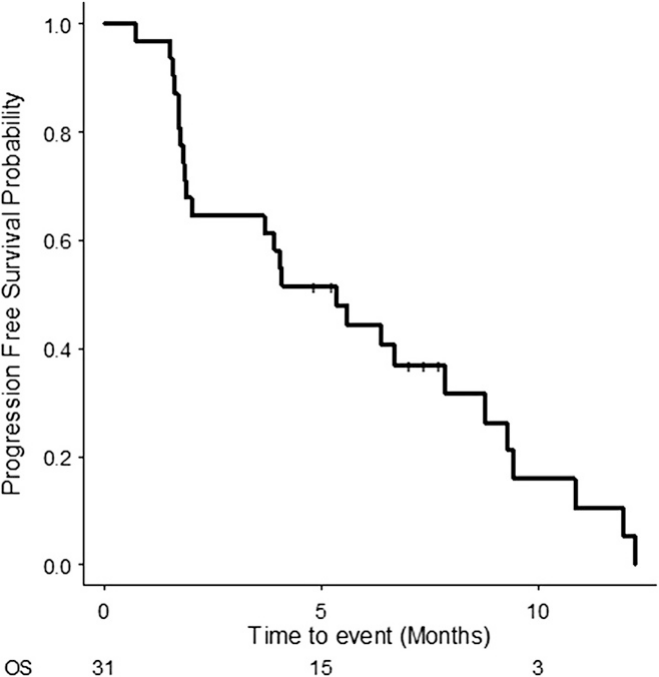
Kaplan–Meier survival curve for progression free survival (PFS) in the intention to treat (ITT) population (*n* = 31). The median PFS was 5.3 months (95 % CI, 2.1–9.3)

**Fig. 3:**
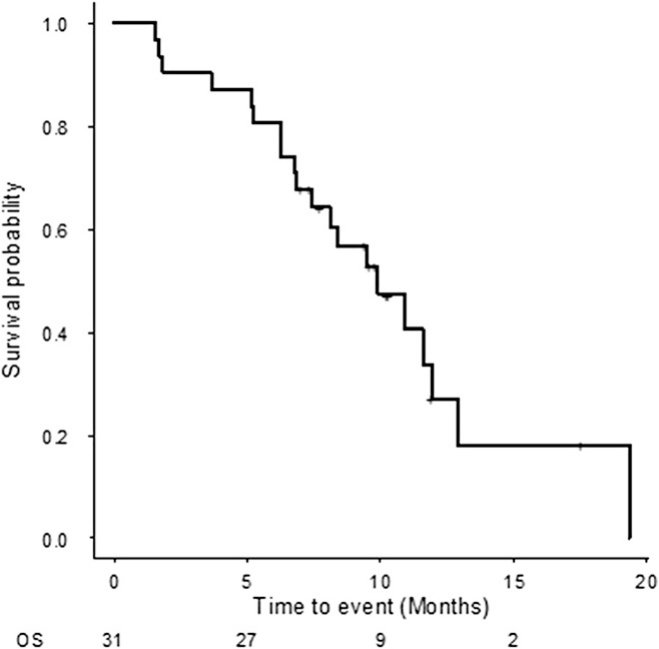
Kaplan-Meier survival curve for overall survival (OS) in the intention to treat (ITT) population (*n* = 31). The median OS was 9.9 months (95 % CI, 7.4-NA)

Three patients (9 %) were not eligible for assessments because of an early termination of the treatment protocol: 2 because of disease progression and 1 because of sudden death, possibly related to treatment. Of the 28 evaluated patients, no patient achieved CR, two patients (7 %) achieved PR, 18 patients (58 %) achieved SD and 8 patients (26 %) showed PD, according to the independent review committee. DCR was 65 % (95 % CI, 48–100 %) and the overall response rate was 7 % (95 % CI, 0.7–22 %) by the ITT analysis. The lower limit of one-sided confidence interval of DCR (45 %) was higher than the predefined null value (22 %); therefore, this study met its primary endpoint. The highest percentage change from baseline in the sum of the longest diameter of the target lesion, as assessed by the central review committee, is shown in Fig. 1. During the trial, 13 patients (42 %) showed some decrease in lesion size compared with the baseline value. The median follow-up period was 11.8 months (range 7.0–20.3 months, interquartile range 9.6–18.2 months) as of June 2014. There were a total of 28 patients with progression and 21 patients who died whilst the median PFS and OS were 5.3 months (95 % CI, 2.1–9.3 months; Fig. 2) and 9.9 months (95 % CI, 7.4 months- NA; Fig. 3), respectively. DCR was similar for both *KRAS-*wild-type (61 %) and *KRAS*-mutant (69 %) statuses, whereas both patients who achieved PR were *KRAS* wild type. Among two patients with PR, one patient had a *BRAF*-mutant tumour with multiple liver metastases from an ascending colon cancer. The patient had primary resistance to capecitabine + oxaliplatin + Bev combination therapy as first-line chemotherapy and irinotecan + cetuximab combination therapy as second-line chemotherapy. This patient had a tumour reduction of 35 % on day 50 and maintained the tumour response until day 126. The patient’s serum tumor markers including carcinoembryonic antigen (CEA) and carbohydrate antigen 19–9 (CA19-9) levels decreased remarkably during the protocol treatment. Median PFS in patients with wild-type and mutant *KRAS* was 5.3 and 3.9 months, and median OS was 10.9 and 9.5 months, respectively.

### Drug delivery

The total number of treatment cycles administered was 299 and the median number of cycles administered was nine (range 1–24, interquartile range 4–13). Dose reduction was required in 16 patients (52 %). The most common causes of dose reduction were grade-3 stomatitis or diarrhea. The relative dose intensity to the planned dose was 78 % for S-1 and LV and 87 % for Bev. Currently, three patients (9 %) remain on the protocol therapy. Twenty-eight patients discontinued treatment: 26 (84 %) because of disease progression and 2 (7 %) because of adverse events. Reasons for discontinuation due to adverse events included infection and sudden death. Sixteen patients have received treatment after the study, including 14 patients being treated with regorafenib, six patients with re-introduction chemotherapy and one patient with a new investigational drug.

### Toxicity and QOL

Toxicity data are summarized in Table 2. Non-hematological toxicities were more common than hematological toxicities. The most common non-hematological toxicities of any grade were stomatitis (74 %), fatigue (74 %), anorexia (68 %) and diarrhea (48 %). The most common grade-3/4 non-hematological toxicity was stomatitis (26 %) and diarrhea (10 %). Eight patients experienced grade-3 stomatitis, which occurred in the first treatment cycle in six patients and in the fourth cycle in two patients. However, this toxicity was reversible with either appropriate treatment, interruption or dose reduction. No patients required hospitalisation and had to terminate the protocol treatment for this reason. Diarrhea was easily managed by using anti-diarrheal drugs and was more responsive than we expected. No grade-4 adverse events of non-hematological toxicity were observed. Hematological toxicities were generally mild and grade-3/4 hematological toxicities were rare. There was a single case of treatment-related death; a 70-year-old male with multiple lung metastases suddenly died on day 9 of the fourth cycle. The cause of death was most likely because of cardiorespiratory failure but was not definitively determined. For QOL, overall mean mobility, self-care, usual activities, pain/discomfort and anxiety/depression subscale scores remained stable from before treatment to after treatment.

**Table 2:** Toxicity (*n* = 31)

	Number of patients, *n* (%)					
	Grade 1	Grade 2	Grade 3	Grade 4	All grades	Grade 3/4
Hematological adverse events						
Anemia	4	4	2	0	10 (32)	2 (6)
Thrombocytopenia	7	1	0	0	8 (26)	0 (0)
Neutropenia	4	1	1	0	6 (19)	1 (3)
Febrile neutropenia	–	–	1	0	1 (3)	1 (3)
Non-hematological adverse events						
Stomatitis	7	8	8	0	23 (74)	8 (26)
Fatigue	13	10	0	–	23 (74)	0 (0)
Anorexia	10	9	2	0	21 (68)	2 (6)
Diarrhea	8	4	3	0	15 (48)	3 (10)
Skin pigmentation	8	4	0	0	12 (39)	0 (0)
Hypertension	7	2	1	0	10 (32)	1 (3)
Nausea	8	1	0	–	9 (29)	0 (0)
Epistaxis	9	0	0	0	9 (29)	0 (0)
Watering eyes	4	1	0	–	5 (16)	0 (0)
Vomiting	3	1	0	0	4 (13)	0 (0)

The most common toxicities of all grade were stomatitis (74 %), fatigue (74 %), anorexia (68 %) and diarrhea (48 %)

The major grade 3–4 toxicities were stomatitis (26 %) and diarrhea (10 %)

## Discussion

In this study, first, we demonstrated SL/Bev has significant clinical activity, yielding 58 % disease stabilisation. This, together with 7 % partial response, resulted in 65 % DCR in heavily pre-treated mCRC. This trial met the pre-specified primary endpoint and also achieved favourable median PFS and OS of 5.3 and 9.9 months, respectively. Furthermore, SL/Bev was generally tolerated and appears to have little impact on QOL.

Several prospective studies have evaluated various cytotoxic and/or targeted agents in the setting of refractory mCRC, and potent new anti-cancer agents have been developed in recent years. The results of large phase-III study of regorafenib, oral multi-kinase inhibitor, showed prolonged survival compared with a placebo in patients with heavily pre-treated mCRC, with a DCR of 41 %, median PFS of 1.9 months and median OS of 6.4 months [15]. In addition, TAS-102 reported encouraging results compared with placebo in randomized phase-III study, which is a new oral nucleoside anti-tumour agent consisting of trifluorothymidine and a thymidine phosphorylase inhibitor [17]. The results showed a DCR of 44.0 %, median time PFS of 2.0 months and median OS of 7.1 months. Compared with the results of previous studies, the present study’s DCR of 65 % for refractory patients was higher and the median OS of 9.9 months was longer. Although, the favourable OS was partly due to post-study anti-cancer treatment; approximately half of the failures (16 of 28) underwent further treatment after stopping protocol therapy. Indeed, a PFS of 5.3 months is much better than that reported previously in salvage settings. Moreover, two patients achieved PR despite resistance to several chemotherapies. These results indicate SL/Bev has individual anti-cancer activity in this heavily pre-treated population.

In this study, SL/Bev showed an acceptable toxicity, considering that it was previously a heavily treated population. The most common adverse events in our study were non-hematological toxicities such as stomatitis and diarrhea. Grade-3/4 stomatitis reported in this study was 26 %, which is higher than that in a previous study for this schedule in patients with untreated mCRC, where stomatitis was reported in only 8.3 % patients [18]. This discrepancy may be ascribed to the fact that patients included in the current study were more heavily treated. Although dose modification was required in 16 of the 31 patients because of mucositis, no discontinuation for this reason was reported, suggesting that mucositis was generally manageable with dose reduction/interruption. Adverse events potentially associated with Bev were well tolerated. Although epistaxis and hypertension were relatively frequent, both symptoms were ≤ grade 2. Moreover, serious adverse events, such as thrombosis and bowel perforation, were not observed. The safety of Bev addition appears to be high. However, it should be noted that one patient died of sudden death possibly related to therapy; this illustrates the need for more caution when treating such a fragile patient population.

The rationale for this combination therapy was based on prior success with S-1 monotherapy against refractory mCRC and the synergistic effects of LV + Bev combination therapy. In a phase-II study, S-1 monotherapy showed promising activity in heavily pre-treated patients with mCRC who had previously failed a regimen containing irinotecan and oxaliplatin. A DCR of 42.9 %, median PFS of 3–4 months and a median OS of 10–13 months were achieved, which suggest that S-1 has potent tumor activity even in 5-FU-refractory mCRC [5]. Several preclinical reports have presented evidence to support the effectiveness of S-1. For example, S-1 showed higher tumor growth inhibition than UFT did in an orthotopic implantation model of colon cancer, and it promoted anti-tumor activity in 5-FU-resistant cancer cell lines [19]. In addition, it has been suggested that DPD inhibition plays a significant role of S-1 in chemo-resistant cancer cells. According to the results of the randomized phase-II/III study, which verify the non-inferiority of IRIS regimen (irinotecan + S-1) to FOLFIRI regimen (fluorouracil + LV + irinotecan) as second-line chemotherapy, IRIS regimen was superior to the FOLFIRI regimen for mCRC patients who previously received an oxaliplatin-based regimen [20]. This difference was explained by the fact that patients previously treated with an oxaliplatin-containing regimen had a higher level of DPD gene expression in the tumor tissue than those not treated with oxaliplatin, and this mechanism was related to the stronger effects of S-1 [21].

In the present study, the enhancement made in the treatment regimen for refractory mCRC was the S-1/LV combination therapy. The addition of LV enhanced the anti-tumor activity of S-1 by TS inhibition. Compared with S-1 monotherapy for previously untreated mCRC, S-1/LV combination therapy demonstrated an improvement in objective response rate from 35 to 57 % and improvement in time to progression from 5.3 to 6.7 months [8]. Moreover, it is reported that acquisition of resistance to 5FU is related to increased TS expression. 5FU-resistant cell lines show increased TS mRNA expression, protein expression, and activity compared with their respective parent cells in *in vitro* and *in vivo* assays [22, 23]. On the basis of this finding, concurrent treatment with LV may have contributed in overcoming resistance to 5FU and yielded some efficacy in the present study.

Little comparative data are available regarding the activity of Bev after second-line therapy. Bev therapy in the later-line setting has been reported in several phase-II studies and in retrospective series [24–29]. According to these reports, Bev does not show a tendency for reduction in tumour size but results in tumour stabilisation and improved survival. However, studies evaluating its effect in later-line treatment of Bev re-introduction are limited. Recently, international multicentre study (ML18147 study) revealed that continuation of Bev after initial tumour progression significantly improved PFS and OS [12]. Similar result was also observed in an Italian multicentre study (BEBYP study), which demonstrated a significant improvement in PFS and OS continuing Bev plus second-line chemotherapy [13]. These results imply disease may still partially depend on VEGF after disease progression and raises the possibility that the angiogenic signal may continue throughout the tumor lifespan. With this speculation, Bev re-introduction may still contribute to enhance anti-tumour activity that has already proved to be resistant. Although we must note that prospective and randomized clinical trials are lacking regarding the role of Bev in chemo-refractory mCRC patients, we could speculate that a combination with Bev provides some efficacy in a heavily treated population.

With regard to the difference in patients in *KRAS* mutation status in this study, no significant difference was observed in either disease stabilization or survival benefit between *KRAS* mutation status. Another finding in this study which is noteworthy, is 1 patient who had *BRAF* mutation achieved PR. *BRAF* mutation is associated with poor prognosis because of more aggressive and rapidly progressing disease and is also predictive of a lack of response to chemotherapy in mCRC. However, in this patient, the size of hepatic metastases remarkably decreased after resistance to all standard chemotherapies. This fact suggests that in patients with *BRAF* mutation, SL/Bev may have potent anti-tumour activity based on some specific effects. A possible explanation for this is the role played by γ-glutamyl hydrolase (GGH) in regulating intracellular folate levels. Low GGH expression is associated with higher folate levels, leading to the enhancement of anti-tumor activity in 5-FU with LV [30]. In the *BRAF*-mutated oncogene, the CpG island methylator phenotype (CIMP+) frequently occurs within gene promoter regions, and CIMP+ is associated with low GGH expression. For this reason, SL/Bev may be more effective in *BRAF*-mutant patients than in *BRAF*-wild-type ones. Although this phenomenon may have occurred by chance, it remains noteworthy.

This study has some limitations. One is that it is a single-arm study design with no control group for comparison. Therefore, we cannot rule out some potential bias, such as the selection of patients with good prognosis. In fact, a majority of patients except two in our study (94 %) had an ECOG PS status of <2. The second limitation is it is unclear whether the efficacy and safety of S-1 in Japanese patients would be similar to those in Western patients. Despite these limitations above, SL/Bev may provide an additional advantage. This regimen considers that no cross-resistance to new anti-cancer agents for refractory mCRC, such as regorafenib and TAS102, exists. Therefore, SL/Bev may provide a therapeutic option even after failure with these agents. To confirm its efficacy, further prospective randomized control trial is necessary to compare SL/Bev with BSC in patient with refractory mCRC.

## Conclusions

In conclusion, SL/Bev showed promising activity in heavily pre-treated patients with mCRC who showed failure of 5-FU, oxaliplatin, irinotecan, Bev and anti-EGFR antibody treatment. A further randomized control trial will be needed to fully evaluate the usefulness of these current findings.

## Additional file

Additional file 1: **Summary of chemotherapies for metastatic colorectal cancer in the various setting.** (PDF 133 kb)

## Abbreviations

Bev: Bevacizumab
BSC: Best supportive care
CA19-9: Carbohydrate antigen 19–9
CEA: Carcinoembryonic antigen
CIMP: CpG island methylator phenotype
CR: Complete remission
DCR: Disease control rate
DPD: Dihydropyrimidine dehydrogenase
ECOG: Eastern Cooperative Oncology Group
EGFR: Epithelial growth factor receptor
GGH: γ-glutamyl hydrolase
LV: Leucovorin
mCRC: Metastatic colorectal cancer
NA: Not available
OS: Overall survival
PD: Progression disease
PFS: Progression-free survival
PR: Partial remission
PS: Performance status
SD: Stable disease
SL: S-1 + oral leucovorin
TS: Thymidylate synthase
VEGF: Vascular endothelial growth factor

## References

[1] National Comprehensive Cancer Network (2014a) NCCN Clinical Practice Guidelines in Oncology: Colon Cancer, version 3.2014. http://www.nccn.org/professionals/physician_gls/f_guidelines.asp. Accessed May 20 2014.

[2] Schmoll HJ, Van Cutsem E, Stein A, Valentini V, Glimelius B, Haustermans K, Nordlinger B, van de Velde CJ, Balmana J, Regula J, Nagtegaal ID, Beets-Tan RG, Arnold D, Ciardiello F, Hoff P, Kerr D, Köhne CH, Labianca R, Price T, Scheithauer W, Sobrero A, Tabernero J, Aderka D, Barroso S, Bodoky G, Douillard JY, EI Ghazaly H, Gallardo J (2012). ESMO Consensus Guidelines for management of patients with colon and rectal cancer. A personalized approach to clinical decision making. Ann Oncol.

[3] Hong YS, Park YS, Lim HY, Lee J, Kim TW, Kim KP, Kim SY, Baek JY, Kim JH, Lee KW, Chung IJ, Cho SH, Lee KH, Shin SJ, Kang HJ, Shin DB, Jo SJ, Lee JW (2012). S-1 plus oxaliplatin versus capecitabine plus oxaliplatin for first-line treatment of patients with metastatic colorectal cancer: a randomised, non-inferiority phase 3 trial. Lancet Oncol.

[4] Yamada Y, Takahari D, Matsumoto H, Baba H, Nakamura M, Yoshida K, Yoshida M, Iwamoto S, Shimada K, Komatsu Y, Sasaki Y, Satoh T, Takahashi K, Mishima H, Muro K, Watanabe M, Sakata Y, Morita S, Shimada Y, Sugihara K (2013). Leucovorin, fluorouracil, and oxaliplatin plus bevacizumab versus S-1 and oxaliplatin plus bevacizumab in patients with metastatic colorectal cancer (SOFT): an open-label, non-inferiority, randomised phase 3 trial. Lancet Oncol.

[5] Jeung HC, Rha SY, Cho BC, Yoo NC, Roh JK, Roh WJ, Chung HC, Ahn JB (2006). A phase II trial of S-1 monotherapy in metastatic colorectal cancer after failure of irinotecan- and oxaliplatin-containing regimens. Br J Cancer.

[6] Thirion P, Michiels S, Pignon JP, Buyse M, Braud AC, Carlson RW, O’Connell M, Sargent P, Piedbois P, Meta-Analysis Group in Cancer (2004). Modulation of fluorouracil by leucovorin in patients with advanced colorectal cancer: an updated meta-analysis. J Clin Oncol.

[7] Douillard JY, Hoff PM, Skillings JR, Eisenberg P, Davidson N, Harper P, Vincent MD, Lembersky BC, Thompson S, Maniero A, Benner SE (2002). Multicenter phase III study of Uracil/Tegafur and oral leucovorin versus fluorouracil and leucovorin in patients with previously untreated metastatic colorectal cancer. J Clin Oncol.

[8] Koizumi W, Boku N, Yamaguchi K, Miyata Y, Sawaki A, Kato T, Toh Y, Hyodo I, Nishina T, Furuhata T, Miyashita K, Okada Y (2010). Phase II study of S-1 plus leucovorin in patients with metastatic colorectal cancer. Ann Oncol.

[9] Hurwitz H, Fehrenbacher L, Novotny W, Cartwright T, Hainsworth J, Heim W, Berlin J, Baron A, Griffing S, Holmgren E, Ferrara N, Fyfe G, Rogers B, Ross R, Kabbinavar F (2004). Bevacizumab plus irinotecan, fluorouracil, and leucovorin for metastatic colorectal cancer. N Engl J Med.

[10] Saltz LB, Clarke S, Díaz-Rubio E, Scheithauer W, Figer A, Wong R, Koski S, Lichinitser M, Yang TS, Rivera F, Couture F, Sirzén F, Cassidy J (2008). Bevacizumab in combination with oxaliplatin-based chemotherapy as first-line therapy in metastatic colorectal cancer: a randomized phase III study. J Clin Oncol.

[11] Giantonio BJ, Catalano PJ, Meropol NJ, O’Dwyer PJ, Mitchell EP, Alberts SR, Schwartz MA, Benson AB, Eastern Cooperative Oncology Group Study E3200 (2007). Bevacizumab in combination with oxaliplatin, fluorouracil, and leucovorin (FOLFOX4) for previously treated metastatic colorectal cancer: results from the Eastern Cooperative Oncology Group Study E3200. J Clin Oncol.

[12] Bennouna J, Sastre J, Arnold D, Österlund P, Greil R, Van Cutsem E, von Moos R, Viéitez JM, Bouché O, Borg C, Steffens CC, Alonso-Orduña V, Schlichting C, Reyes-Rivera I, Bendahmane B, André T, Kubicka S, ML18147 Study Investigators (2013). Continuation of bevacizumab after first progression in metastatic colorectal cancer (ML18147): a randomised phase 3 trial. Lancet Oncol.

[13] Masi G, Loupakis F, Salvatore L, Cremolini C, Fornaro L, Schirripa M, Granetto C, Miraglio E, Costanzo FD, Antonuzzo L, Marcucci L, Barbara C, Boni C, Banzi M, Chiara S, Garbarino D, Valsuani C, Bonetti A, Boni L, Falcone A (2013). Second-line chemotherapy (CT) with or without bevacizumab (BV) in metastatic colorectal cancer (mCRC) patients (pts) who progressed to a first-line treatment containing BV: Updated results of the phase III “BEBYP” trial by the Gruppo Oncologico Nord Ovest (GONO) [abstract]. J Clin Oncol.

[14] Oken MM, Creech RH, Tormey DC (1982). Toxicity and response criteria of the Eastern Cooperative Oncology Group. Am J Clin Oncol.

[15] Grothey A, Van Cutsem E, Sobrero A, Siena S, Falcone A, Ychou M, Humblet Y, Bouché O, Mineur L, Barone C, Adenis A, Tabernero J, Yoshino T, Lenz HJ, Goldberg RM, Sargent DJ, Cihon F, Cupit L, Wagner A, Laurent D, CORRECT Study Group (2013). Regorafenib monotherapy for previously treated metastatic colorectal cancer (CORRECT): an international, multicentre, randomised, placebo-controlled, phase 3 trial. Lancet.

[16] Yoshino T, Mizunuma N, Yamazaki K, Nishina T, Komatsu Y, Baba H, Tsuji A, Yamaguchi K, Muro K, Sugimoto N, Tsuji Y, Moriwaki T, Esaki T, Hamada C, Tanase T, Ohtsu A (2012). TAS-102 monotherapy for pretreated metastatic colorectal cancer: a double-blind, randomised, placebo-controlled phase 2 trial. Lancet Oncol.

[17] Yoshino T, Mayer R, Falcone A (2014). Results of a multicenter, randomized, double-blind, phase III study of TAS-102 vs. placebo, with best supportive care (BSC), in patients (pts) with metastatic colorectal cancer (mCRC) refractory to standard therapies (RECOURSE) [abstract]. Ann Oncol.

[18] Denda T, Li J, Xu R, Xu J, Ikejiri K, Shen L, Toh Y, Shimada K, Kato T, Baba H (2012). Phase II study of S-1 plus leucovorin (a new 1-week treatment regimen followed by a 1-week rest period) in patients with untreated metastatic colorectal cancer in Japan and China [abstract]. J Clin Oncol.

[19] Shirasaka T, Nakano K, Takechi T, Satake H, Uchida J, Fujioka A, Saito H, Okabe H, Oyama K, Takeda S, Unemi N, Fukushima M (1996). Antitumor activity of 1 M tegafur-0.4 M 5-chloro-2,4-dihydropyridine-1 M potassium oxonate (S-1) against human colon carcinoma orthotopically implanted into nude rats. Cancer Res.

[20] Muro K, Boku N, Shimada Y, Tsuji A, Sameshima S, Baba H, Satoh T, Denda T, Ina K, Nishina T, Yamaguchi K, Takiuchi H, Esaki T, Tokunaga S, Kuwano H, Komatsu Y, Watanabe M, Hyodo I, Morita S, Sugihara K (2010). Irinotecan plus S-1 (IRIS) versus fluorouracil and folinic acid plus irinotecan (FOLFIRI) as second-line chemotherapy for metastatic colorectal cancer: a randomised phase 2/3 non-inferiority study (FIRIS study). Lancet Oncol.

[21] Baba H, Watanabe M, Okabe H, Miyamoto Y, Sakamoto Y, Baba Y, Iwatsuki M, Chikamoto A, Beppu T (2012). Upregulation of ERCC1 and DPD expressions after oxaliplatin-based first-line chemotherapy for metastatic colorectal cancer. Br J Cancer.

[22] Fukushima M, Fujioka A, Uchida J, Nakagawa F, Takechi T (2001). Thymidylate synthase (TS) and ribonucleotide reductase (RNR) may be involved in acquired resistance to 5-fluorouracil (5-FU) in human cancer xenografts in vivo. Eur J Cancer.

[23] Murakami Y, Kazuno H, Emura T, Tsujimoto H, Suzuki N, Fukushima M (2000). Different mechanisms of acquired resistance to fluorinated pyrimidines in human colorectal cancer cells. Int J Oncol.

[24] Chen HX, Mooney M, Boron M, Vena D, Mosby K, Grochow L, Jaffe C, Rubinstein L, Zwiebel J, Kaplan RS (2006). Phase II multicenter trial of bevacizumab plus fluorouracil and leucovorin in patients with advanced refractory colorectal cancer: an NCI Treatment Referral Center Trial TRC-0301. J Clin Oncol.

[25] Geva R, Vecchione L, Tejpar S, Piessevaux H, Van Cutsem E, Prenen H (2013). Bevacizumab plus chemotherapy as salvage treatment in chemorefractory patients with metastatic colorectal cancer. Onco Targets Ther.

[26] Kang BW, Kim TW, Lee JL, Ryu MH, Chang HM, Yu CS, Kim JC, Kim JH, Kang YK, Lee JS (2009). Bevacizumab plus FOLFIRI or FOLFOX as third-line or later treatment in patients with metastatic colorectal cancer after failure of 5-fluorouracil, irinotecan, and oxaliplatin: a retrospective analysis. Med Oncol.

[27] Lam KO, Lee VH, Liu RK, Leung TW, Kwong DL (2013). Bevacizumab-containing regimens after cetuximab failure in KRAS wild-type metastatic colorectal carcinoma. Oncol Lett.

[28] Lièvre A, Samalin E, Mitry E, Assenat E, Boyer-Gestin C, Lepère C, Bachet JB, Portales F, Vaillant JN, Ychou M, Rougier P (2009). Bevacizumab plus FOLFIRI or FOLFOX in chemotherapy-refractory patients with metastatic colorectal cancer: a retrospective study. BMC Cancer.

[29] Park LC, Lee HS, Shin SH, Park SJ, Park MI, Oh SY, Kwon HC, Baek JH, Choi YJ, Kang MJ, Kim YS (2012). Bevacizumab as a second- or later-line of treatment for metastatic colorectal cancer. World J Gastroenterol.

[30] Kawakami K, Ooyama A, Ruszkiewicz A, Jin M, Watanabe G, Moore J, Oka T, Iacopetta B, Minamoto T (2008). Low expression of gamma-glutamyl hydrolase mRNA in primary colorectal cancer with the CpG island methylator phenotype. Br J Cancer.

