# Supplementary material for: A single-arm phase II trial of combined chemotherapy with S-1, oral leucovorin, and bevacizumab in heavily pre-treated patients with metastatic colorectal cancer
Source: BMC Cancer. 2015 Aug 27;15:601. doi: 10.1186/s12885-015-1606-1 (PMC4550068; doi:10.1186/s12885-015-1606-1)
Supplement: Additional file 1: — Summary of chemotherapies for metastatic colorectal cancer in the various setting. (PDF 133 kb) [file 12885_2015_1606_MOESM1_ESM.pdf]

Additional file 1

Treatment	KRAS	RR (%)	mPFS (months)	mOS (months)
**first-line**				
FOLFOX/FOLFIRI/XELOX + Bev [1]	-	47	9.4	21.3
FOLFOX/FOLFIRI + C-mab [2]	WT	61	7.7	22.8
FOLFOX/FOLFIRI + P-mab [3]	WT	55	9.6	23.9
**second-line**				
FOLFOX/FOLFIRI + Bev [4]	-	22.7	7.3	12.9
FOLFIRI + Aflibercept [5]	-	19.8	6.9	13.5
FOLFIRI + P-mab [6]	WT	35	5.9	14.5
**further-line**				
Irinotecan + C-mab [7]	WT	22.9	4.1	8.6
C-mab [8]	WT	19.8	4.4	10.0
P-mab [8]	WT	22.0	4.1	10.4
Regorafenib [9]	-	1.0	1.9	6.6
TAS102 [10]	-	1.6	2.0	7.1

Abbreviations; Bev, bevacizumab; C-mab, cetuximab; DCR, disease control rate; FOLFIRI, infusional 5-fluorouracil/bolus+ folnic acid+ irinotecan; FOLFOX, infusional 5-fluorouracil/bolus+ folnic acid+ oxaliplatin; mOS, median overall survival; mPFS, median progression-free survival; NA, not available; P-mab, panitumumab; RR, response rate; WT, wild type; XELOX, capecitabine+ oxaliplatin.

Additional references

1. Saltz LB, Clarke S, Díaz-Rubio E, Scheithauer W, Figer A, Wong R, Koski S, Lichinitser M, Yang TS, Rivera F, Couture F, Sirzén F, Cassidy J: **Bevacizumab in combination with oxaliplatin-based chemotherapy as first-line therapy in metastatic colorectal cancer: a randomized phase III study.** *J Clin Oncol* 2008, **26**:2013-9.
2. Bokemeyer C, Bondarenko I, Makhson A, Hartmann JT, Aparicio J, de Braud F, Donea S, Ludwig H, Schuch G, Stroh C, Loos AH, Zubel A, Koralewski P: **Fluorouracil, leucovorin, and oxaliplatin with and without cetuximab in the first-line treatment of metastatic colorectal cancer.** *J Clin Oncol* 2009, **27**:663-71.
3. Douillard JY, Siena S, Cassidy J, Tabernero J, Burkes R, Barugel M, Humblet Y, Bodoky G, Cunningham D, Jassem J, Rivera F, Kocákova I, Ruff P, Błasińska-Morawiec M, Šmakal M, Canon JL, Rother M, Oliner KS, Wolf M, Gansert J: **Randomized, phase III trial of panitumumab with infusional fluorouracil, leucovorin, and oxaliplatin (FOLFOX4) versus FOLFOX4 alone as first-line treatment in patients with previously untreated metastatic colorectal cancer: the PRIME study.** *J Clin Oncol* 2010, **28**:4697-705.
4. Giantonio BJ, Catalano PJ, Meropol NJ, O'Dwyer PJ, Mitchell EP, Alberts SR, Schwartz MA, Benson AB 3rd; Eastern Cooperative Oncology Group Study E3200: **Bevacizumab in combination with oxaliplatin, fluorouracil, and leucovorin (FOLFOX4) for previously treated metastatic colorectal cancer: results from the Eastern Cooperative Oncology Group Study E3200.** *J Clin Oncol* 2007, **25**:1539-44.

5. Van Cutsem E, Tabernero J, Lakomy R, Prenen H, Prausová J, Macarulla T, Ruff P, van Hazel GA, Moiseyenko V, Ferry D, McKendrick J, Polikoff J, Tellier A, Castan R, Allegra C: **Addition of aflibercept to fluorouracil, leucovorin, and irinotecan improves survival in a phase III randomized trial in patients with metastatic colorectal cancer previously treated with an oxaliplatin-based regimen.** *J Clin Oncol* 2012, **30**:3499-506.
6. Peeters M, Price TJ, Cervantes A, Sobrero AF, Ducreux M, Hotko Y, André T, Chan E, Lordick F, Punt CJ, Strickland AH, Wilson G, Ciuleanu TE, Roman L, Van Cutsem E, Tzekova V, Collins S, Oliner KS, Rong A, Gansert J: **Randomized phase III study of panitumumab with fluorouracil, leucovorin, and irinotecan (FOLFIRI) compared with FOLFIRI alone as second-line treatment in patients with metastatic colorectal cancer.** *J Clin Oncol* 2010, **28**:4706-13.
7. Cunningham D, Humblet Y, Siena S, Khayat D, Bleiberg H, Santoro A, Bets D, Mueser M, Harstrick A, Verslype C, Chau I, Van Cutsem E: **Cetuximab monotherapy and cetuximab plus irinotecan in irinotecan-refractory metastatic colorectal cancer.** *N Engl J Med* 2004, **351**:337-45.
8. Price TJ, Peeters M, Kim TW, Li J, Cascinu S, Ruff P, Suresh AS, Thomas A, Tjulandin S, Zhang K, Murugappan S, Sidhu R: **Panitumumab versus cetuximab in patients with chemotherapy-refractory wild-type KRAS exon 2 metastatic colorectal cancer (ASPECCT): a randomised, multicentre, open-label, non-inferiority phase 3 study.** *Lancet Oncol* 2014, **15**:569-79.
9. Grothey A, Van Cutsem E, Sobrero A, Siena S, Falcone A, Ychou M, Humblet Y, Bouché O, Mineur L, Barone C, Adenis A, Tabernero J, Yoshino T, Lenz HJ, Goldberg RM, Sargent DJ, Cihon F, Cupit L, Wagner A, Laurent

D; CORRECT Study Group: **Regorafenib monotherapy for previously treated metastatic colorectal cancer (CORRECT): an international, multicentre, randomised, placebo-controlled, phase 3 trial.** *Lancet* 2013, **381**:303-12.

10. Yoshino T, Mayer R, Falcone. **Results of a multicenter, randomized, double-blind, phase III study of TAS-102 vs. placebo, with best supportive care (BSC), in patients (pts) with metastatic colorectal cancer (mCRC) refractory to standard therapies (RECOURSE) [abstract].** *Ann Oncol* 2014, **25**:s2.
